# Autophagy and multidrug resistance in cancer

**DOI:** 10.1186/s40880-017-0219-2

**Published:** 2017-06-24

**Authors:** Ying-Jie Li, Yu-He Lei, Nan Yao, Chen-Ran Wang, Nan Hu, Wen-Cai Ye, Dong-Mei Zhang, Zhe-Sheng Chen

**Affiliations:** 1 Institute of Traditional Chinese Medicine & Natural Products, College of Pharmacy, Jinan University, Guangzhou, 510632 Guangdong P. R. China; 2 Department of Pharmaceutical Sciences, College of Pharmacy and Health Sciences, St. John’s University, Queens, NY 11439 USA

**Keywords:** Autophagy, Drug resistance, Neoplasms, Cell survival, Cell death

## Abstract

Multidrug resistance (MDR) occurs frequently after long-term chemotherapy, resulting in refractory cancer and tumor recurrence. Therefore, combatting MDR is an important issue. Autophagy, a self-degradative system, universally arises during the treatment of sensitive and MDR cancer. Autophagy can be a double-edged sword for MDR tumors: it participates in the development of MDR and protects cancer cells from chemotherapeutics but can also kill MDR cancer cells in which apoptosis pathways are inactive. Autophagy induced by anticancer drugs could also activate apoptosis signaling pathways in MDR cells, facilitating MDR reversal. Therefore, research on the regulation of autophagy to combat MDR is expanding and is becoming increasingly important. We summarize advanced studies of autophagy in MDR tumors, including the variable role of autophagy in MDR cancer cells.

## Background

Biosynthesis and degradation are two processes involved in the maintenance of metabolic homeostasis, which is the basis of all biological activities. The major protein degradation systems include the ubiquitin–proteasome pathway (targeting short-lived and misfolded proteins) and the lysosome-autophagy system (targeting long-lived macromolecular complexes and organelles) [1, 2]. Accumulating evidence indicates that autophagy plays a vital role in maintaining homeostasis in cells, and deficient autophagy seriously impacts embryonic differentiation. Autophagy is also closely related to the development of many diseases, including Alzheimer’s disease, cancer, and microorganism infection [3, 4]. Autophagy is a highly conserved cellular process in which cytoplasmic materials are degraded and recycled to maintain energy homeostasis. Autophagy can be classified as macroautophagy, microautophagy, and chaperone-mediated autophagy [5]. Given that current researches primarily focus on macroautophagy and the mechanisms are more clearly established than other types of autophagy, the term “autophagy” is commonly used to refer to macroautophagy.

Autophagy is a successive process which is initiated by the formation of an isolation membrane called phagophore. The phagophore is often seen as a thin cisterna with a clear lumen and is the structure that recruits autophagy-related proteins to induce autophagy. This lipid-based membrane then elongates and creates a complete, closed, double-membrane structure containing damaged organelles or long-lived proteins. Ultimately, autolysosomes are formed to degrade the contents, recycling amino acids, fatty acids, and nucleotides to maintain energetic homeostasis and viability [4, 6]. Autophagy occurs frequently during tumorigenesis and cancer chemotherapy. In general, constructive autophagy protects cancer cells during chemotherapy, leading to cancer drug resistance and refractory cancer [7].

Multidrug resistance (MDR) is another refractory outcome of chemotherapy and is defined as the resistance of cancer cells to multiple chemotherapeutic drugs with different structures and mechanisms of action [8]. MDR is a major cause of chemotherapy failure and responsible for increasing cancer-related mortality. Interestingly, recent mechanistic investigations have demonstrated that autophagy pathways are involved in the development of MDR [9]. Recent studies have explored approaches of using autophagy to hijack MDR cancer cells during anticancer therapy, but the mechanisms underlying the relationship between autophagy and MDR have not been fully studied.

## Mechanisms of autophagy

Autophagy is a highly conserved cellular process. Approximately 30 autophagy-related genes (*Atgs*) in yeast and many mammalian genetic homologs have been identified [10]. *Atgs* are essential in responses to microenvironmental stresses such as hypoxia, heat stress, and accumulation of reactive oxygen species (ROS). Autophagy proceeds in successive stages, including initiation of phagophore assembly, autophagosomal formation, and lysosomal fusion [11, 12]. Here, we briefly discuss some primary pathways that regulate autophagy.

### Phagophore assembly

The formation of autophagosomes begins with the expansion of the membrane core. The serine/threonine-protein kinase ULK1 complex containing unc-51-like autophagy activating kinase 1 (ULK1), Atg13, and focal adhesion kinase (FAK) family kinase-interacting protein 200 (FIP200) is at the most upstream position during autophagosome formation [13, 14]. Autophagosomal membrane contains a high level of phosphatidylinositol 3-phosphate (PI3P) in comparison to other types of membrane within the cell [15]. The formation of the autophagosomal membrane is regulated by class III PtdIns3K complexes containing Vacuolar protein sorting-associated protein 34 (Vps34), Vps15, Beclin1, and Atg14, which regulates the process that generates PtdIns(3)P-rich membranes [16]. Activating molecule in BECN1-regulated autophagy protein 1-deleted in liver cancer 1 (Ambra1-DLC1) released from the dynein motor complex acts as a cofactor of Beclin1 in a ULK1-dependent manner and is essential to autophagosome [17]. The autophagosomal membrane is thought to be derived from endoplasmic reticulum-Golgi [18]. The phagophore may be built up from the endoplasmic reticulum-mitochondria contact site [19]. Other compartments, such as the endosomes and the plasma membrane, also contribute to the formation of autophagosomes [20].

### Autophagosome formation and maturation 3

Following autophagy initiation by the formation of phagophores, double-membrane autophagosomes (which load degradative cargos) are assembled under the control of the Atg12 conjugation system. In this system, the E1-like enzyme Atg7 and E2-like enzyme Atg10 jointly catalyze the formation of the Atg12–Atg5 complex, which is covalently conjugated [21]. Ultimately, the Atg12–Atg5–Atg16 (L1) complex is formed and directly binds membranes and constructs autophagosomes [22]. The complex is efficient for the microtubule-associated protein 1 light chain 3 (LC3) conjugation system. LC3 is first conjugated with lipid phosphatidylethanolamine (PE). During the conversion, Atg4 plays a role in lipoxidating LC3 to LC3-I and exposes the C-terminal glycine of LC3 for the subsequent conjugation of PE [23]. PE is conjugated to the C-terminal glycine of LC3-I, and this conjugation needs to be catalyzed by the E1-like enzyme Atg7 and the E2-like enzyme Atg3 [24].

### Autolysosome degradation

The autophagosome is degraded by docking and fusing with a lysosome to construct an autolysosome. The autophagosomal membrane is conjugated with LC3-PE. During fusion with the lysosome, the outer membrane is cleaved and recycled by Atg4, while LC3-PE associated with the inner membrane is degraded by lysosomal proteases along with the cargo of the autophagosome, thus recycling amino acids, fatty acids, and nucleotides [25].

### Core regulator of autophagy

The mechanistic target of rapamycin (mTOR), which regulates cell growth and survival, is the central modulator of autophagy regulation. As an environmental sensor, mTOR responds to intracellular microenvironmental changes including amino acids and glucose, as well as extracellular stresses such as agent treatments, hypoxia, and ultraviolet radiation. mTOR is active under nutrient-rich conditions and inhibits autophagy and protein degradation. By contrast, mTOR is inactive under nutrient-poor conditions: dephosphorylated ULK1 dissociates from the mTOR complex and then phosphorylates Atg13 and RB1-inducible coiled-coil (1RB1CC1/FIP200), thus triggering autophagy [26]. Inactivation of mammalian target of rapamycin complex 1 (mTORC1) by amino acid starvation can activate Atg14-containing type III phosphatidylinositol (PtdIns) 3-kinase (PIK3C3/VPS34) and induce autophagy both in vitro and in vivo [27]. The PIK3C3/VPS34 inhibitor SAR405 inhibits autophagy induced by mTOR inhibition, further indicating a crucial role of kinase regulation by mTOR in regulating autophagy [28]. In addition, mTOR can regulate autolysosome reformation by directly activating PIK3C3, leading to autolysosomal tubule sorting and lysosome regeneration [29].

## Mechanisms of MDR

**Fig. 1:**
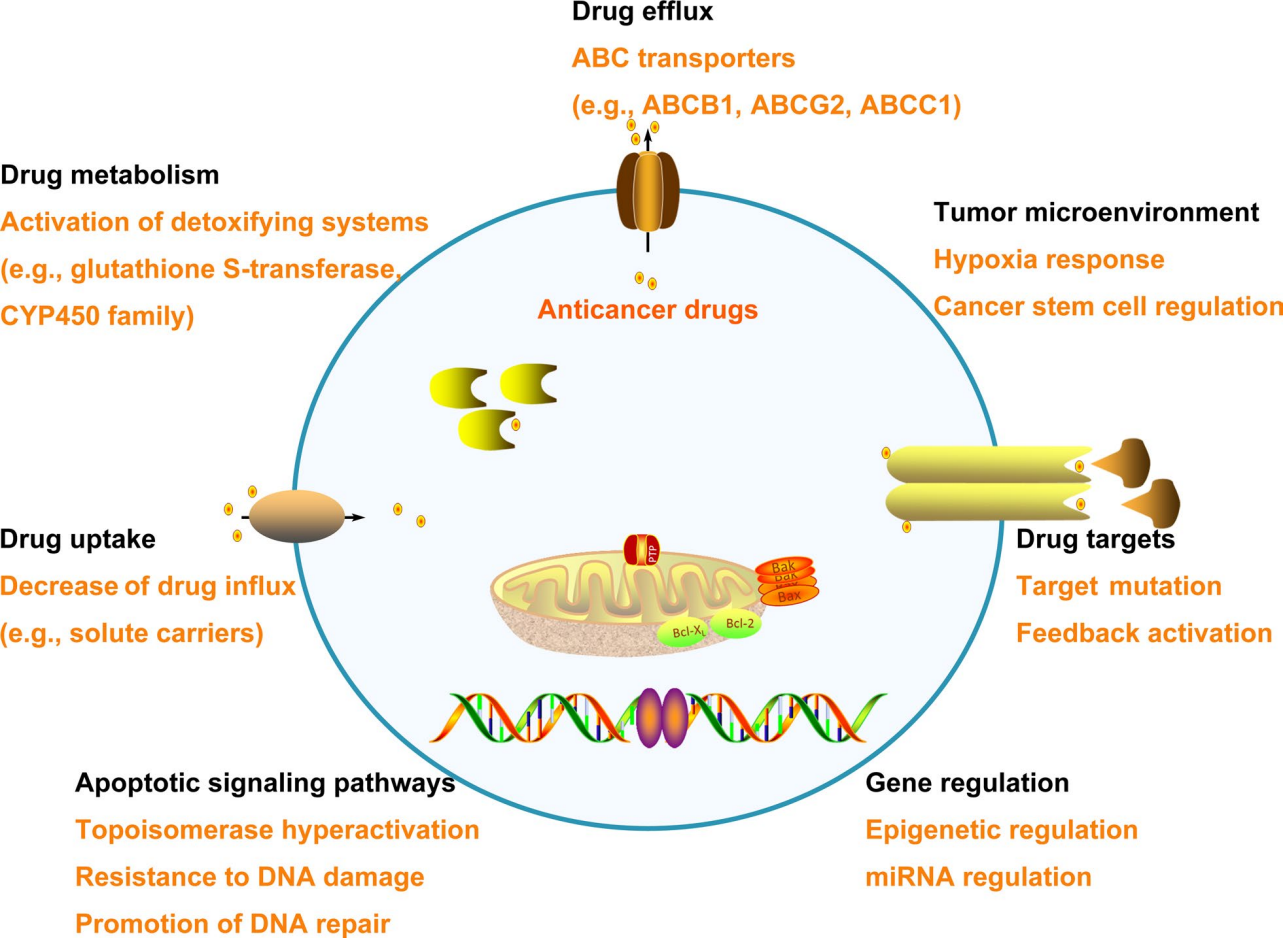
Complicated mechanisms of multidrug resistance (MDR) in tumor. The main mechanism of MDR is overexpressing ATP-binding cassette (ABC) transporters to increase drug efflux, resulting in a decrease in intracellular drug concentration. Other mechanisms of MDR are reducing drug uptake by influx transporters, boosting drug metabolism, blocking apoptotic signaling pathways, elevating adaptability by epigenetic regulation and microRNA regulation, mutation in drug targets or feedback activation of other targets and signaling pathways, and change of tumor microenvironment. ABCB1, ATP-binding cassette subfamily B member 1; ABCG2, ATP-binding cassette subfamily G member 2; ABCC1, ATP-binding cassette subfamily C member 1; CYP450, cytochrome P450

Mechanisms of MDR can be divided into seven categories: (1) increasing drug efflux by membrane transporters, with ATP-binding cassette (ABC) transporters as the main transporters [30]; (2) reducing drug uptake by influx transporters, such as solute carriers [31]; (3) boosting drug metabolism, including elimination by glutathione S-transferase and cytochrome P450 enzymes [32, 33]; (4) blocking apoptotic signaling pathways due to change in the expression level of B cell lymphoma (BCL) family proteins or mutations in the p53 pathway [34, 35]; (5) elevating adaptability by epigenetic regulation and miRNA regulation [36, 37]; (6) mutation in drug targets or feedback activation of other targets and signaling pathways [38]; and (7) chemoresistance induced by changes in the microenvironment, such as hypoxia response and cancer stem cell regulation [39, 40] (Fig. 1). Cellular-based resistant mechanisms are further classified into transporter-based classical MDR phenotypes and non-classical MDR phenotypes.

The ABC superfamily contains 49 different types of transporters and can be classified into seven subfamilies from ABC-A to ABC-G based on sequence similarities and structural organization [9]. Among them, P-glycoprotein (P-gp/ABCB1), multidrug-resistant protein 1 (MRP1/ABCC1), breast cancer resistant protein (BCRP/ABCG2/MXR/ABCP), and multidrug-resistant protein 10 (ABCC10/MRP7) transporters frequently drive chemosensitive cancers to MDR [41]. Human ABCB1 was the first identified ABC transporter. Overexpression of ABCB1 contributes to resistance against a wide variety of chemotherapeutic drugs. ABCC1 also leads to resistance to a wide range of anticancer drugs, and extensive evidence indicates that resistance of cancer cells to mitoxantrone, saquinavir, epipodophyllotoxins, and anthracyclines is mediated by ABCC1 [42–45]. The ABCG2 transporter is primarily expressed in breast cancer, colon cancer, gastric cancer, small cell lung cancer, and ovarian cancer [31]. ABC transporters are recognized as chief culprits in the development of MDR. Therapies continue to be developed with the goal of blocking or inactivating ABC transporters to increase the concentration of anticancer drugs within cells [46].

## The relationship between autophagy and MDR

Chemotherapeutic agents that kill cancer cells primarily act by inducing apoptosis. Deficient apoptosis has been proposed to contribute to the development of MDR. Thus, alternative anticancer drugs that can directly induce apoptosis of MDR cancer cells would be valuable. However, the inactivation of apoptosis pathways adds to the complexity and difficulty of the development of such drugs. Importantly, MDR is inevitable following prolonged exposure to a new agent. Accordingly, other types of programmed cell death in MDR cells have attracted increasing attention, with autophagy emerging as a promising candidate.

Complex and controversial evidence indicating a role of autophagy in tumorigenesis has emerged in recent years. Autophagy can play a protective role against cancer by eliminating damaged organelles and recycling degradation products in normal cells. Paradoxically, excessive autophagy can devote cancer cells to “autophagic cell death” or “type II programmed cell death.” Thus, autophagy induced by metabolic and therapeutic stresses can have a pro-death or pro-survival role. Autophagy also plays dual roles in tumorigenesis, tumor progression, and resistance of cancer cells to chemotherapy [47]. Autophagy can be activated as a protective mechanism to mediate MDR during treatment. Thus, the inhibition of autophagy can re-sensitize resistant cancer cells and enhance the effect of chemotherapeutic agents. However, autophagy may also induce autophagic cell death, which differs from type I programmed cell death (apoptosis). Thus, autophagy can be used to promote the efficacy of treatment on MDR cancer if applied properly, and the role of autophagy in MDR must be clarified.

### Autophagy as a cytoprotective mechanism mediating MDR

Because ABC transporters are associated with MDR, agents that modulate ABC transporters have been advocated to be developed as targets for chemotherapeutic drugs to overcome MDR. However, current ABC transporter modulators have not been as effective in the clinic as expected. Furthermore, MDR is an extremely complex phenotype. Recent studies have focused on clarifying the connection between autophagy and MDR based on clinical data. The expression level of *ABCB1* was positively correlated with *LC3*, *Beclin1*, *Rictor* expression levels and negatively correlated with *Raptor* expression level in tumor samples from colorectal cancer patients surviving 5 years [48], strongly indicating that autophagy is related to the development and progression of cancer and MDR in clinical settings.

#### Autophagy promotes the development of MDR

Substantial evidence demonstrates that MDR develops after autophagy. The enhanced autophagy levels detected in patients with poor prognosis indicate that the presence of autophagy may catalyze the development of MDR. Yang et al. [49] demonstrated that S100 calcium-binding protein A8 (S100A8) is involved in the development of MDR by regulating autophagy in leukemia cells. Adriamycin and vincristine treatment can up-regulate the expression of S100A8, which is required for the formation of the Beclin1–Class III phosphatidylinositol 3-kinase (PI3KC3) complex [49, 50]. MDR may be mediated by high-mobility group box 1 (HMGB1), which promotes protective autophagy in response to anticancer agents. HMGB1 translocates from the nucleus to the cytoplasm and promotes the formation of Beclin1–PI3KC3 complexes by activating the mitogen-activated protein kinase (MAPK)/extracellular signal-regulated kinase (ERK) signaling pathway [51]. Similarly, Fan et al. [52] demonstrated that peptidylarginine deiminase IV may lead to MDR in hepatocellular carcinoma cells by inducing autophagy as a protective function.

MicroRNAs (miRNAs) can target *Atgs* and are considered as important modulators in MDR cancer cells. Xu et al. [53] confirmed that miR-199a-5p is down-regulated after cisplatin-based chemotherapy, resulting in cisplatin resistance via activation of autophagy. By contrast, miR-181a targets *Atg5* and inhibits autophagy, thus enhancing the cytotoxicity of cisplatin in cisplatin-selective MDR SGC7901/CDDP cells in vitro and in vivo [54].

Recent studies have revealed that autophagy triggered by chemotherapeutics may facilitate resistance of cancer cells to paclitaxel [55], tamoxifen [56], epirubicin [57], or trastuzumab [58]. Sun et al. [57] found that epirubicin induced autophagy not only in MCF-7 cells but also in epirubicin-resistant MCF-7er cells. The induction of autophagy in MCF-7er cells may defend against epirubicin-mediated apoptosis, act as a pro-survival factor, and lead to deficient apoptosis [57]. Sun et al. [59] further studied two epirubicin-resistant cell lines, MCF-7er and SK-BR-3er, which overexpress ABCB1 and are simultaneously insensitive to paclitaxel and vinorelbine. Their study revealed that paclitaxel and vinorelbine induce autophagy, protecting MDR cells from apoptosis [59]. Although the expression of ABCB1 is independent of autophagy, the induction of MDR by autophagy cannot be ignored.

#### Autophagy inhibition facilitates the efficiency of chemotherapy in MDR cancer

As MDR-promoted autophagy is well-documented, new therapeutic strategies incorporating a combination of autophagy inhibitors have been proposed (Table 1). Inhibition of autophagy via genetic silencing of *Atgs* such as *Beclin1*, *Atg5*, *Atg7*, and *Atg12* sensitizes MDR cells to therapeutic agents [73–75]. For example, chemoresistant cancer cell line SGC7901/VCR develops upon exposure to vincristine and exhibits increased autophagy, which is regulated by *Atg12* and high-mobility group box 2 (*HMGB2)*. Overexpression of miR-23b-3p, which targets *Atg12* and *HMGB2*, sensitizes chemoresistant cells to multiple chemotherapeutics such as vincristine, 5-fluorouracil, and cisplatin in vitro and can restore the sensitivity of MDR cells to chemotherapy in vivo [60]. Furthermore, inhibition of autophagy by small interfering RNAs (siRNAs) targeting *Atg12* and *HMGB2* or by the autophagy inhibitor chloroquine (CQ) also sensitizes SGC7901/VCR cells to chemotherapeutics [60]. This finding indicates that vincristine-based MDR is related to autophagy. In response to 5-fluorouracil, cisplatin, or other chemotherapeutic drugs, some types of MDR cells exhibit protective autophagy and rapidly recover after the removal of chemotherapeutic agents [61]. Basal autophagic flux is higher in the anthracycline-resistant lines MDA-MB-231-R8 and SUM159PT-R75 than in their respective parent lines MDA-MB-231 and SUM159PT [76]. Furthermore, *Atg5/Atg7* knockdown as well as pharmacologic inhibition of autophagy with CQ or hydroxychloroquine (HCQ) significantly reduce viability of both epirubicin-sensitive and epirubicin-resistant triple-negative breast cancer cells [76]. These findings suggest that autophagy responds to anthracycline-induced MDR and protects MDR cells from injury.

**Table 1:** Recent studies on the pro-survival role of autophagy in multidrug-resistant (MDR) cancer

Intervention for tumor treatment	Cell line	Method(s) to study autophagy	References
miR-23b-3p	Vincristine-resistant SGC7901	CQ, siRNAs (*Atg12*, *HMGB2*)	[60]
5-FU	Drug-resistant esophageal cancer cells	3-MA, siRNAs (*Beclin1*, *Atg7*)	[61]
Epirubicin	Epirubicin-resistant MDA-MB-231	CQ	[62]
Ursolic acid	*PTEN*-deficient PC3	3-MA, siRNAs (*Atg5*, *Beclin1*)	[63]
FTY720	Cisplatin-resistant ovarian cancer cells	siRNAs (*Beclin1*, *LC3*), Baf A1	[64]
PI-103	*PTEN*-deficient glioma cell lines	siRNA (*Atg5*), Baf A1, 3-MA	[65]
PP2	Ras-NIH3T3/Mdr	3-MA	[66]
SAHA	Imatinib-resistant CML cells	CQ	[67]
B-raf inhibitors	B-Raf inhibitor-resistant melanoma cells	HCQ	[68]
Cisplatin	Cisplatin-resistant A549/DDP	3-MA	[69]
Docetaxel	Adriamycin-resistant MCF-7	CQ	[70]
Doxorubicin	Adriamycin-resistant MCF-7	CQ	[71]
Vincristine	VCR-resistant ovarian carcinoma SKVCR	3-MA, CQ	[72]

5-FU, 5-fluorouracil; FTY720, 2-amino-2-[2-(4-octylphenyl)]-1,3-propanediol hydrochloride; PP2, 4-amino-5-(4-chloro-phenyl)-7-(*t*-butyl)pyrazolo[3,4-*d*]pyrimidine; SAHA, suberoylanilide hydroxamic acid; *PTEN*, phosphatase and tensin homologue; CML, chronic myelogenous leukemia; VCR, vincristine resistance; CQ, chloroquine; siRNA, small interfering RNA; *Atg*, autophagy-related gene; *HMGB2*, high-mobility group box 2; 3-MA, 3-methyladenine; Baf A1, bafilomycin A1; LC3, protein 1 light chain 3; HCQ, hydroxychloroquine

### Inducing autophagic cell death overcomes MDR

Autophagy can also play a pro-death role and trigger autophagic cell death in apoptosis-deficient MDR cells. Several studies have sought to identify a novel anticancer agent that effectively kills MDR cancer cells by inducing excessive autophagy (Table 2). Suberoylanilide hydroxamic acid (SAHA), a newly developed prototype histone deacetylase inhibitor, induces autophagic cell death in tamoxifen-resistant MCF-7 breast cancer cells and significantly suppresses tumor growth [77]. Two lipophilic tanshinones, cryptotanshinone and dihydrotanshinone, also inhibit the growth of apoptosis-resistant colon cancer cells by inducing autophagic cell death and *p53*-independent cytotoxicity [78]. The bisindolic alkaloid voacamine induces autophagy, which causes MDR tumor cell death [75]. The resistance of the leukemic cell line K562 to edelfosine can be reversed by edelfosine lipid nanoparticles, which induce caspase-independent and autophagic cell death [79]. These results indicate that autophagic cell death can be induced in MDR cells as an alternative cell death mechanism when the cells fail to undergo apoptosis.

**Table 2:** Recent studies on the pro-death role of autophagy in MDR cancer

Intervention for tumor treatment	Cell line	Method(s) to study autophagy	References
SAHA	Tamoxifen-resistant MCF-7	3-MA	[77]
Tanshinones	Apoptosis-resistant SW620	3-MA	[78]
Edelfosine lipid nanoparticles	Edelfosine-resistant leukemic K562	Starvation, staurosporine	[79]
GMI protein	Multidrug-resistant lung cancer cells	CQ	[80]
NVP-BEZ235	Cisplatin-resistant urothelial cancer cells	3-MA	[81]
Cisplatin	Cisplatin-resistant H460	3-MA, trifluoperazine	[82]
RAD001	Apoptotic deficient H460	3-MA, siRNAs (*Atg5*, *Beclin1*)	[83]
Isoliquiritigenin	Adriamycin-resistant MCF-7	3-MA, CQ	[84]
*p53* plasmids	Multidrug-resistant SKVCR	3-MA	[85]
Hernandezine	Apoptosis-resistant cell lines	*Atg7*-knockout, 3-MA	[86]
HTCC-MNPs	Drug-resistant SGC7901	3-MA	[87]
Quinacrine	Chemoresistant ovarian cancer cells	Baf A1	[88]

SAHA, suberoylanilide hydroxamic acid; GMI, Ganoderma microsporum immunomodulatory; HTCC-MNPs, *N*-[(2-hydroxy-3-trimethylammonium)propyl] chitosan chloride/alginate-encapsulated Fe_3_O_4_ magnetic nanoparticle; 3-MA, 3-methyladenine; Baf A1, bafilomycin A1; siRNA, small interfering RNA; *Atg*, autophagy-related gene; CQ, chloroquine

Several signaling pathways contribute to autophagic cell death in MDR cancer cells (Table 1). For instance, the AMP-activated protein kinase (AMPK)-protein kinase B (Akt)-mTOR pathway is critical to the regulation of autophagic cell death. A Ganoderma microsporum immunomodulatory (GMI) protein inhibits the phosphorylation of Akt and the 70 kDa S6 protein kinase (p70S6K) in the A549 lung cancer sub-cell lines A549/D16 and A549/V16, inducing autophagy and overcoming MDR in lung cancer [80]. Similarly, the dual phosphoinositide 3-kinase (PI3K) and mTOR inhibitor NVP-BEZ235 suppresses the proliferation of cisplatin-resistant urothelial cancer cells by activating autophagic flux independent of apoptotic cell death [81]. Other cellular signaling cascades may also be involved in autophagic cell death in MDR cells. For example, a human single-chain fragment variable, HW1, kills tumor necrosis factor-related apoptosis-inducing ligand (TRAIL)-resistant cancer cells by inducing autophagic cell death, which can be inhibited by autophagy inhibitors. HW1-mediated autophagic cell death occurs primarily through the c-Jun NH2-terminal kinase pathway [89].

### Autophagy facilitates cell death in apoptosis-deficient MDR cancer

In apoptosis-deficient MDR cells, an adaptive response of autophagy may aggravate MDR cancer resistance to chemotherapeutics. However, under certain conditions, autophagy can be a scavenger in apoptosis-blocked signaling pathways, sensitizing MDR tumors to apoptosis. Feroniellin A, a novel furanocoumarin derives from *Feroniella lucida*, elicits autophagy dependent on the mTOR/Beclin1/Atg5 pathway in etoposide-resistant, ABCB1-overexpressed A549 cells. Interestingly, inhibition of autophagy by *Beclin1* siRNA could eliminate Feroniellin A-induced apoptosis. Moreover, further stimulation of autophagy by rapamycin accelerates Feroniellin A-induced apoptosis [90]. Coincidentally, autophagy induced by metformin could also assists TRAIL-mediated apoptosis in TRAIL-resistant lung adenocarcinoma. Metformin is found to down-regulate cellular FADD-like IL-1β-converting enzyme (FLICE)-inhibitory protein (c-FLIP) and markedly enhance autophagic flux, ultimately facilitates apoptosis triggered by TRAIL [91].

### Autophagy mediates chemosensitization

**Fig. 2:**
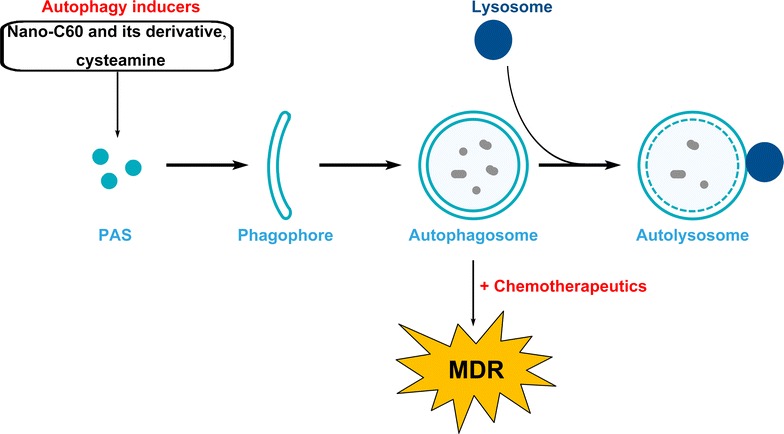
Autophagy chemosensitizes MDR cancer cells to chemotherapeutics. A novel role of autophagy induced by certain autophagy inducers such as cysteamine, the nanocrystal of underivatized fullerene C60 (nano-C60) and its derivative was confirmed. Autophagy triggered by those inducers could sensitive MDR cancer cells to chemotherapeutics. PAS, pre-autophagosomal structure

A plenty of studies have revealed that autophagy, the chemotherapeutics’ appendant, is a patron of MDR cancer cells, consequently harass sensitive effect of MDR-reversal agents. Conversely, emerging evidence arises that the increase in autophagy upon some agents could facilitate MDR reversal (Fig. 2). For example, the nanocrystal of underivatized fullerene C60 (nano-C60) exhibits potential anticancer property to several neoplasms in vitro [92]. Nano-C60 is capable of triggering oxidative stress and then induces autophagy which is enhanced by photoactivation. In particular, autophagy induced by nano-C60 is able to sensitive drug-resistant MCF-7 cancer cells. The chemosensitizing effect of nano-C60 can be dampened after autophagy inhibition by a ROS scavenger, *N*-acetyl-l-cysteine. Furthermore, the chemosensitizing effect of nano-C60 depends on *Atg5* and is vanished in *Atg5* ^−/−^ cells and *Atg5* siRNA-treated cells [93]. In addition, a derivative of nano-C60 is also capable to vulnerable doxorubicin-resistant MCF-7 cells to doxorubicin by modulating autophagy, and its activity is much greater than that of nano-C60 [94]. Similarly, cysteamine-elicited autophagy could also reverse resistance of adriamycin-resistant MCF-7/ADR cells to doxorubicin in vitro and in vivo [95]. Although the exact molecular mechanisms remain blurred, those results still strongly highlight a novel biological function of autophagy in MDR reversal strategy.

## Conclusions

In recent years, a large body of evidence has indicated that autophagy plays dual roles in MDR. The pro-death or pro-survival roles of autophagy are highly dependent on the tumor type and treatment characteristics. Autophagy protects MDR cancer cells from apoptosis and promotes resistance to chemotherapy treatment, and inhibition of autophagy may sensitize MDR cells to anticancer drugs. The combination of autophagy inhibitors with cytotoxic drugs is highly anticipated. Excitingly, CQ and its derivative HCQ, in combination with several anticancer drugs, have been approved to augment cytotoxicity and to sensitize refractory cancers. They are expected to be used in the battlefield of MDR tumor. On the other hand, autophagic cell death which is induced by autophagy inducers could directly bypass apoptosis and ultimately eliminate MDR cells. It represents a new battle line in the fight against MDR cancer. On the other battleground, emerging evidence demystifies that autophagy is a strong propulsor to sensitize apoptosis-resistant MDR cells to anticancer drugs and reverse MDR. It shows a novel biological function of autophagy in MDR cancer cells and will enable the development of promising strategies to overcome MDR. Although the exact mechanisms of the interaction between autophagy and MDR reversal remain obscure, it provides us a vast research space to elucidate the mysteries.
